# First-born siblings show better second language skills than later born siblings

**DOI:** 10.3389/fpsyg.2015.00705

**Published:** 2015-06-03

**Authors:** Karin Keller, Larissa M. Troesch, Alexander Grob

**Affiliations:** ^1^Department of Psychology, University of BaselBasel, Switzerland,; ^2^Department of Psychology and Human Development, UCL Institute of Education, University College LondonLondon, UK

**Keywords:** birth order, sibling, second language acquisition, language proficiency, bilingualism

## Abstract

We examined the extent to which three sibling structure variables *number of siblings, birth order*, and *presence of an older sibling at school age* are linked to the second language skills of bilingual children. The research questions were tested using an ethnically heterogeneous sample of 1209 bilingual children with German as a second language. Controlling for children’s age, sex, nationality, number of children’s books at home, family language and parental German language skills, hierarchical regression analyses showed an inverse relationship between the number of siblings and second language skills: the more siblings a child had, the lower was his/her second language proficiency. This relationship was mediated by attendance in early education institutions. Moreover, first-born siblings showed better second language skills than later born siblings. The current study revealed that the resource dilution model, i.e., the decrease in resources for every additional sibling, holds for second language acquisition. Moreover, the results indicate that bilingual children from families with several children benefit from access to early education institutions.

## Introduction

Despite the constant decline in number of children in families, the ideal of the two-child family still persists (Testa, 2012) and it is generally assumed that siblings mutually enrich and learn from one another (Howe and Recchia, 2006). In the case of families from immigrant backgrounds, the assumption is furthermore that older siblings play an important role in integrating the family into the host culture and constitute a facilitator particularly to the language of the host country (Cooper et al., 1999; Shin, 2002).

Language skills, which are the focus of the present study, cover semantic, syntactic, morphologic and pragmatic facets (Saxton, 2010) and are considered as a crucial prerequisite to be successful in and after school. Studies indicated that there is a gap between language skills of immigrant and non-immigrant children starting in early childhood, which does not close even after years of schooling (e.g., Oller and Eilers, 2002). Local language oftentimes implies a second language for immigrant children. Second language skills are acquired in a broad range of developmental contexts that include interactions with educators in early education institutions, with children and adults in the neighborhood as well as interactions within the family (Hoff, 2013). Research regarding familial contexts in the scope of second language acquisition has been sparse so far with existing studies mainly focusing on parental influences (e.g., Becker, 2010). However, empirical evidence of whether and how sibling structure variables are associated with second language skills of children is still pending.

Thus, the present study examines the effect of sibling structure variables on the second language skills of immigrant children. In particular, we investigated whether classic models of sibling structure variables such as the *resource dilution model* can be applied to second language acquisition. These research questions are addressed in a large sample of bilingual children in Switzerland.

### The Resource Dilution Model

Educational studies of the past 50 years report that only children and children with fewer siblings achieve better grades at school and have a higher level of education than individuals from families with many children (e.g., Blau and Duncan, 1967; Park, 2008). The resource dilution model explains this inverse relationship between number of siblings and number of years in education with a decrease in parental resources (Blake, 1981). This model is based on the assumption that parental resources are finite and have to be shared between children within a family. Every additional sibling means a reduction in the share allocated to each child, thus reducing one of the foundations of their intellectual development. According to Blake (1981), parental resources include material resources, extra-familial learning opportunities, and parental attention, intervention and teaching. The strength and the pattern of the relationship vary depending on the type of resource (Downey, 1995, 2001). Some resources, such as a stock of books for example, can be shared between several siblings without any significant reduction in their value. By contrast, financial resources, invested by parents in a child’s extracurricular education for example, appear to be more vulnerable to the number of siblings (Downey, 1995, 2001; Steelman et al., 2002). According to the resource dilution model, parental resources available do not decline linearly with every additional child. Rather, the decline in parental resources as the number of children within a family increases comes closest to the theoretical equation *y* = 1/*x* (Downey, 1995), where *x* represents the total number of children in the family and *y* represents the parental resources available such as financial resources for early education institutions.

### Effect of Number of Siblings

The effects of sibship size become particularly clear when measuring school success and education indicators such as number of school years (Blake, 1981; Iacovou, 2007), but they can also be seen in studies on standardized intelligence and language measures (Belmont and Marolla, 1973; Polit and Falbo, 1988; Steelman et al., 2002; Sundet et al., 2010). A comparison of non-verbal and verbal measures in Polit and Falbo’s (1988) review revealed a greater vulnerability in the field of language.

Research into sibling effects on second language acquisition is sparse. It therefore remains unclear whether and in which way the number of siblings is linked to the second language skills of bilingual children, and whether the effects of diminishing parental resources can be seen in a way that is analogous to first language development. Given a greater degree of direct interaction between parents and child in families with fewer children (Jones and Adamson, 1987), we assume that children from smaller families benefit more from parents’ knowledge of the local language (when extant) or indirectly gain a better foundation for acquiring a second language through better support in the first language (e.g., reading picture books together; Verhoeven, 1994; Uchikoshi, 2006). Based on Downey’s (1995) finding that financial resources have a particularly strong tendency to dilute and the knowledge that children pose a risk of poverty (European Commission, 2008), we assume that families with many children have fewer financial resources to provide educational opportunities outside the family and therefore, the likelihood of a child attending early education institutions is lower.

As early education institutions constitute key places for the second language acquisition of immigrant children (Aukrust and Rydland, 2011; Niklas et al., 2011; Halle et al., 2012), we suppose that limited access to these institutions has a negative effect on the second language development, i.e., attendance in an early education institution is assumed to mediate the relationship between the number of siblings and second language skills. This mediational effect is all the more plausible as access to the early childcare system in Switzerland and some other European countries is limited and parental costs for institutional childcare are high (OECD, 2013).

Although knowledge to date suggests that an inverse relationship exists between the number of siblings and second language skills among bilingual children according to the resource dilution model, no evidence exists confirming this assumption. Ortiz (2009), for example, examined 747 Latino preschool children in the USA assessing English language skills by means of a standardized receptive language test. In Ortiz’s study, contrary to the author’s expectations, there was no association between the number of children and knowledge of English language skills.

### Effect of Birth Order

During their early years of life — or at least their first year — first-born children do not have to share parental attention and financial resources for early education institutions with their younger siblings. Thus, based on the resource dilution model it can be assumed that first-born children have an advantage over later-born siblings during childhood. Various studies have shown that there are differences favoring first-born children both in regard to the onset of speech and in regard to level of language skills (Pine, 1995; Zambrana et al., 2012). In Fenson et al.’s (1994) large-scale study first-born children showed greater abilities in word production in both infancy and toddlerhood. The Swedish study of Berglund et al. (2005) with over one thousand 18-months-old children revealed significant negative effects of birth order on both the production and the comprehension of words. However, the strength of these effects was minimal, with an explained variance of 1.7% in vocabulary production and 0.5% in vocabulary comprehension respectively. Based on a study with pairs of siblings, Pine (1995) showed that first-born children achieve the 50-word milestone roughly a month earlier than their second-born siblings. However, no significant differences were found for the 100-word milestone some months later. Differences in the amount and the kind of parental input were assumed to be the reason for the differences between first- and later-born siblings. Studies showed that first-born children are read to more often than later born children (Raikes et al., 2006; Westerlund and Lagerberg, 2008), that these children receive more linguistic input from their mothers, and that the children are more often explicitly encouraged to express themselves (Jones and Adamson, 1987; Hoff-Ginsberg, 1998).

Although some studies revealed a negative effect of birth order, there are also studies, that found no differences in standardized language tests or even suggested that later-born children are at an advantage (Jenkins and Astington, 1996; Oshima-Takane et al., 1996; Hoff-Ginsberg, 1998; Bornstein et al., 2004; Westerlund and Lagerberg, 2008). For example, Oshima-Takane et al. (1996) showed that later-born children used personal pronouns earlier, which the authors attributed to more frequent triadic interactions with the mother and the elder sibling. Hoff-Ginsberg (1998) reported disadvantages in vocabulary and grammar in later-born children, but also noted a developmental advantage in conversational skills.

The extent to which birth order is associated with second language skills among immigrant children remains unclear. On the one hand, it is conceivable that mechanisms similar to those in monolingual children are at work, and that higher second language skills can be expected in first-born children during their first few years due to their situation as only children. On the other hand, it has been repeatedly reported that older siblings constitute a facilitator to the local language (e.g., Shin, 2002) and that second-born children accordingly have been expected to experience more favorable conditions of acquisition and have better second language skills. This advantage might be particularly true for children with an older sibling in school age. To become an effective language partner, older siblings need to possess a certain level of second language skills. In school age, children improve their second language skills und thus pose a significant source of language exposure to the younger sibling (Bridges and Hoff, 2014). Moreover, at school, older siblings learn the importance of local language skills and bring that knowledge into the home. Younger siblings might profit from the insights and second language skills of their older siblings and thus, improve their local language skills (Wong Fillmore, 1991; Shin, 2002).

These two approaches — the resource dilution model as well as elder siblings as facilitators for the second language acquisition of their younger siblings — explain the issue on different levels and are not mutually exclusive. Thus it is conceivable that while the processes of interaction between siblings benefit learning, the arrival of an additional sibling changes the relationship constellation and the financial situation of a family to such an extent that the second-born child is placed at a developmental disadvantage.

To date, evidence for both lines of arguments is sparse and mixed. Ortiz (2009) assumed better second language skills for later-born children, but failed to demonstrate evidence in a group of Latino preschool children in the USA. No effect of birth order on knowledge of the second language emerged either in David and Wei’s (2008) longitudinal study with 13 French- and English-speaking children nor in Caspar and Leyendecker’s (2011) study with 88 Turkish-German-speaking children. Bridges and Hoff (2014) also examined older siblings’ influence on language skills in a total of 87 English–Spanish bilingual toddlers in the USA assessing English and Spanish language skills using caregiver report measures. In contrast to the previous findings, in their study, children with an older sibling showed more advanced English language skills. Moreover, bilingual children with an older school-aged sibling were more skilled in English.

In sum, the current state of research is marked first by a lack of studies on sibling structure variables among immigrant children and, second, by mixed results. Thus, further studies are needed to shed light on the significance of siblings on second language skills of bilingual children.

### The Current Study

The current study examined the extent to which three sibling structure variables, — i.e., number of siblings, birth order and presence of an elder sibling at school age — are connected to the second language skills of bilingual children, and whether the resource dilution model can be adopted to the second language acquisition of immigrant children. *First*, we postulated an inverse relationship between the number of siblings and second language skills. *Second*, we examined whether attending an early education institution mediates the relationship between number of siblings and second language skills referring to the resource dilution model (Blake, 1981; Downey, 1995, 2001). *Third*, we examined whether, in analogy to studies on early first language development, better second language skills can be expected of first compared to later-born children (e.g., Zambrana et al., 2012), or whether, conversely, later-born children benefit from their older siblings and show higher levels of second language skills (e.g., Shin, 2002; Zambrana et al., 2012). To complement the third hypothesis, we examined whether the effect of the birth order depends of the age gap to the older sibling. We assumed that later-born children have higher levels of second language skills if the older sibling is already at school age and can thus be expected to possess better German and better communication skills (Bridges and Hoff, 2014).

## Materials and Methods

### Procedure

The data of this study stem from the Basel research project Zweitsprache [Second Language]. One goal of the project Zweitsprache was to develop a parental questionnaire to assess German language skills of immigrant children. Another goal was to examine the educational and care situation of children from immigrant backgrounds and analyses their developmental trajectory from pre-school to first grade. The sample was recruited in Basel, a city with 194,000 inhabitants in the German-speaking part of Switzerland.

The study was audited by the Ethic Review Committee of the City and the County of Basel (EKBB) and approved as ethically unobjectionable.

The study was based on a parental questionnaire sent to all families in Basel with a child between two-and-a-half and 3 years of age in the years 2009, 2010, 2011, and 2012. The questionnaire was employed three-and-a-half years before the children started school. The Basel-Stadt statistical office provided the addresses to which the questionnaire was posted. Parents were able to either complete the questionnaire themselves at home or to take part in an information event, where trained assistants with a Bachelor degree in psychology and/or intercultural intermediaries assisted in filling out the questionnaires. Of the 4,739 conveyed forms, 2,608 were returned, 131 could not be delivered, and 2,000 have not been sent back.

### Sample

The present study included only children with German as a second language and children with German as a bilingual first language. The total sample consisted of 1,209 children (47.7% girls) aged between 32 and 45 months (*M* = 38.9, *SD* = 3.7). The children originated from Switzerland (39.5%), former Yugoslavia (10.8%), Turkey (9.9%), Italy (5.3%), Portugal (4.1%), India (3.0%), Germany (2.6%), Sri Lanka (2.5%), Spain (2.2%), UK (2.2%), and 62 further countries with frequencies <2%.

A majority of the children (81%), 11.3% of mothers and 12.8% of fathers were born in Switzerland. The average length of the stay in Switzerland for parents born abroad was 10.45 years for mothers and 12.84 years for fathers, respectively. In 31.5% of families, only the native language was spoken at home, in 26.4% the native language was usually spoken, in 38.5% the native language and German in equal measure, in 5.0% usually German and in 1.3% only German was spoken.

### Measures

#### German Language Skills

Language skills contain semantic, syntactic, morphologic, and pragmatic facets and are regarded as a complex system of rules (e.g., Saxton, 2010). In the current study German language skills were assessed using the standardized parental questionnaire DaZ-E (Keller and Grob, 2013), which covers receptive as well as productive aspects of language skills with special emphasis on semantics and pragmatics. The questionnaire consists of 17 items. For example, parents had to answer the following questions: *“How often does your child say something in German* (e.g., *to parents, other children, relatives etc.)?”* [0 = never, 1 = rarely, 2 = sometimes, 3 = often] or “*Does your child understand the following questions in German?” “Wo ist das Fenster?”* [English translation: “Where is the window?”], *“Was ist dein Lieblingsessen?”* [English translation: “What’s your favorite food?”], *“Wie gross bist du?”* [English translation: “How tall are you?”], [0 = no/I cannot rate, 1 = yes]. The items add up to a sum score that is linearly transformed to a scale with a range from 0 (*low*) to 10 (*high*).

The questionnaire exists in Albanian, Bosnian/Serbian/Croatian, German, English, French, Italian, Portuguese, Spanish, Tamil, and Turkish. The different language versions of the questionnaire have been checked for measurement invariance (*RMSEA* = 0.042; Δ*RMSEA* = 0.009; *CFI* = 0.944; Δ*CFI* = 0.008). The questionnaire versions have a reliability of Cronbach’s α of 0.92 to 0.97, a concurrent validity with the Language Development Test SETK-2 (Grimm, 2000) of 0.84, and a test–retest reliability of 0.95 over a period of 4 months (Keller and Grob, 2013).

#### Sibling Structure Variables

Sibling structure variables were assessed at the same time as language skills. Parents provided information about the individuals living in the same household and on the years of birth of any siblings. Thirty-nine percent (*n* = 472) of the sample were only children at the time of the survey, 42.7% (*n* = 516) had one sibling, 13.3% (*n* = 161) two siblings, and 5.0% (*n* = 60) three or more siblings (**Table 1**). The variable *number of siblings* was transformed according to the formula of the resource dilution model -1/*x* (*x* = number of children; Downey, 2001). In order to examine the number of children at which unfavorable effects can be expected to set in, we formed three dummy variables: dummy 1: *no siblings* (= 1) versus *at least one sibling* (= 2); dummy 2: *a maximum of one sibling* (= 1) versus *more than one sibling* (= 2); dummy 3: *a maximum of two siblings* (= 1) versus more than two siblings (= 2).

**Table 1:** Descriptives, mean, and standard deviation of German language skills.

			Language skills^a^	
	*n*	%	*M*	*SD*
**Sib size**				
Only child	472	39.0	4.70	3.27
Two children	516	42.7	4.71	3.28
Three children	161	13.3	4.79	3.23
Four or more children	60	5.0	4.18	2.59
**Birth order**				
First-born	677	56.0	4.78	3.35
Later-born	532	44.0	4.58	3.09
**Older sibling at school age**				
No older sibling at school age	817	67.6	4.81	3.31
Older sibling at school age	392	32.4	4.43	3.06

*^a^Sum scores of the German language questionnaire DaZ-E (Keller and Grob, 2013).*

Regarding the variable birth order, a distinction was made between first-born children (*n* = 677; 56.0%) and later-born children (*n* = 532; 44.0%). Only and first-born children were both included in the category *first-born children*, as comparable intelligence and language scores can be expected according to Polit and Falbo’s (1988) review. 10.8% of the children had at least one sibling that was 1–3 years older, 19.2% had one or more siblings 3–6 years older, 10.5% had one or more siblings 6–9 years older, 6.6% of the children had at least one sibling that was 9–12 years older and 5.9% had at least one sibling that was 12 or more years older.

The variable children with *an older sibling at school age* constituted a subcategory of the category later-born children and referred to children with at least one older sibling with an age gap of 3 or more years. Based on the Swiss school enrolment system, which has a cut-off date, it was possible to deduce that these were children with a sibling at school age (*n* = 392; 32.4%). Sixty-eight percent of the sample (*n* = 817) had no older sibling at school age.

#### Early Education Institution

In the current study, an early education institution is defined as an institution that offers childcare for children before kindergarten entry. In the city of Basel, kindergarten is mandatory and starts at age 4. There are different forms of early educational care such as playgroups or daycare centers. These forms vary in educational concepts as well as opening hours.

Attendance in an early education institution was assessed using parental questionnaires. Parents stated the name of the institution and the weekly length of care in numbers of hours. The average length of care, which served as a mediator in this study, was 10.7 h per week (*SD* = 13.1, range: 0–55 h). The medium length of two to three half-days per week in the sample corresponds to the length of care expected in Switzerland, but is clearly lower than the average hours of care in the USA (31 h/week) and other European countries such as France (31 h/week) or Germany (23 h/week; OECD, 2013, 2014).

#### Control Variables

The information about the children’s sex, age, and nationality was provided by the Basel-Stadt statistical office. The variable nationality was divided according to national languages into 1 = countries where German is the national language and 2 = countries where German is not the national language. The mother’s German skills and the father’s German skills were each measured using an item with a four-point scale in the parental questionnaire (1 = *nonexistent*, 4 = *well*). The control variable family language was ascertained using the question *Does your family speak predominantly German at home or another language?* (1 = *Exclusively German*, 5 = *Exclusively another language*; Keller and Grob, 2013). As a proxy for the home language and linguistic environment the number of children’s books in the household was recorded (*1 = up to 10; 2 = up to 20; 3 = up to 30; 4 = more than 30*).

#### Statistical Procedure

First, we calculated measures of descriptive statistics. Second, in order to examine the effect of three sibling structure variables, — i.e., number of siblings, birth order, and presence of an elder sibling at school age — on German language skills, we conducted hierarchical regression analyses. In each hierarchical regression analysis, the variable German skills was the dependent variable and, in a first step, the control variables sex, age, nationality, number of children’s books, family language, German skills of the mother and German skills of the father were entered, which are important predictors for second language skills (e.g., Becker, 2010). In a second step, the sibling structure variables were added (hypotheses 1 and 3).

The second hypothesis focused on the mediation effect of the length of attendance in an early education institution on the relationship between the number of siblings and German skills. In order to test a mediation effect, the data has to meet the following precondition (a) the effect of the independent variable (in this case: number of siblings) on the mediator (in this case: length of attendance in an early education institution) and (b) the effect of the mediator on the dependent variable (German skills) need to be statistically significant (Baron and Kenny, 1986). In case these preconditions are met, the mediator has to significantly reduce the effect of the independent variable on the dependant variable. One statistical procedure to test whether the mediator significantly reduces the effect of the independent variable on the dependant variable is the Sobel test. It tests whether the indirect effect of the independent variable on the dependent variable through the mediator variable is significant (Baron and Kenny, 1986).

## Results

Descriptive statistics of German skills are displayed in **Table 1**. The bivariate correlations of number of siblings, birth order and presence of an older sibling at school age resulted in correlations with high effects (*r* = 0.63–0.78), which was expected given the conceptual overlap between these three dimensions. Thus the likelihood of an older sibling at school age is greater for children with a higher number of siblings; indeed, it is only possible for later-born children to have older siblings of school age.

The first hypothesis on the effect of the number of siblings was tested using a hierarchical regression analysis with German skills (second language of the children) as a dependent variable. In the first step and in all subsequent analyses, the control variables considered were: sex, age, nationality, number of children’s books, family language, German skills of the mother, and German skills of the father. The second step was to include the target variable number of siblings in the model. This variable had been transformed previously into -1/*x* according to the resource dilution model (Downey, 1995, 2001). The eight variables included in the model explained 38% of the variance in German skills [*R^2^* = 0.379, *F*(8,1208) = 93.32, *p* < 0.001]. In line with our hypothesis, the variable number of siblings produced a significant negative effect on German skills [Δ*R^2^* = 0.002, Δ*F*(1,1200) = 4.61, *p* < 0.05, β = -0.05; **Table 2**]. The more siblings a child had, the lower his/her German skills. However, the variable number of siblings explained only 0.2%. In a further analysis intended to test the number of children at which unfavorable effects can be expected to set in, the number of siblings was included in the model using three dummy variables. Again, in a first step the control variables were included in the hierarchical regression analysis. In the second step, the three dummy variables were included. The dummy variable *no siblings versus at least one sibling* produced a significant effect (β = -0.05, *p* < 0.05, one-tailed), whereas the other two dummy variables *a maximum of one sibling versus more than one sibling* (β = -0.00, *p* = 0.92) and *a maximum of two siblings versus more than two siblings* (β = -0.01, *p* = 0.83) were unable to explain any additional variance in German skills.

**Table 2:** Effect of number of siblings and length of attendance in an early education institution on German language skills.

	German language skills								
	Model 1						Model 2^a^		
Predictors	Step 1			Step 2					
	**β**	***T***	***p***	**β**	***T***	***p***	**β**	***T***	***p***

Constant		-1.85	0.07		-2.32	<0.05		-1.90	0.06
Sex	0.02	0.66	0.51	0.01	0.61	0.54	0.02	1.08	0.28
Age	0.15	6.63	<0.001	0.16	6.80	<0.001	0.13	6.67	<0.001
National language	-0.03	-1.13	0.26	-0.03	-1.09	0.28	-0.04	-0.77	0.08
Number of children’s books	0.36	15.55	<0.001	0.36	15.62	<0.001	0.22	10.30	<0.001
Family language	-0.28	-10.69	<0.001	-0.28	-10.73	<0.001	-0.29	-12.85	<0.001
Mothers’ German language skills	0.22	8.14	<0.001	0.22	8.13	<0.001	0.17	7.50	<0.001
Fathers’ German language skills	0.07	2.75	<0.01	0.07	2.91	<0.01	0.13	5.71	<0.001
Number of siblings				-0.05	-2.15	<0.05	-0.01	-0.39	0.70
Length of attendance							0.42	19.48	<0.001
Δ*R^2^*						0.002			0.149
_c_*_orr_R^2^* (Total)	.		0.378			0.379			0.535

*^a^Step 2 of the hierarchical regression analysis including control variables and Length of attendance; Sex: 1 = male, 2 = female; National language: 1 = countries where German is the national language, 2 = countries where German is not the national language; family language: 1 = exclusively German, 5 = exclusively another language.*

The second hypothesis aimed to test the mediation effect of the length of attendance in an early education institution on the relationship between the number of siblings and second language skills. A regression analysis following the procedure of Baron and Kenny (1986) was applied. Again, the variables sex, age, nationality, number of children’s books, family language, and parental German skills were entered first as control variables. The preconditions of significant effects of (a) the independent variable *number of siblings* on the mediator *length of attendance in an early education institution* [Δ*R^2^* = 0.010, Δ*F*(1,1183) = 13.91, *p* < 0.01, β = -0.10] and (b) the mediator on the dependent variable German skills [Δ*R^2^* = 0.151, Δ*F*(1,1181) = 386.13, *p* < 0.001, β = 0.42] were met. I.e., the more siblings a child had, the fewer hours the child attended an early education institution and the more hours a child attended an early education institution, the better his/her German skills. Taking the variable length of attendance into account, 54% of the variance in German skills was explained [*R^2^* = 0.535, *F*(9,1188) = 152.75, *p* < 0.001]. The variable length of attendance itself explained 15% of the variance [Δ*R^2^* = 0.149, Δ*F*(1,1180) = 379.64, *p* < 0.001, β = 0.42; **Table 2**]. By taking the mediator into consideration, the aforementioned direct effect of the number of siblings on German skills was reduced by 84% [Δ*R^2^* < 0.001, Δ*F*(1,1180) = 0.15, *p* = 0.70, β = -0.01]. The Sobel test resulted in a significant effect (*z* = 3.50, *p* < 0.001). Thus the mediation of the length of attendance in an education institution was confirmed in line with the resource dilution model.

The third hypothesis focused on the effect of birth order on second language skills and was also tested using a hierarchical regression analysis. The control variables were entered in a first step and the independent variable *birth order* (1 = first-born, 2 = later-born) in a second step. The variables included in the model explained 38% of the total variance [*R^2^* = 0.381, *F*(8,1208) = 94.05, *p* < 0.001]. The predictor birth order had a significant negative effect on German skills [Δ*R^2^* = 0.004, Δ*F*(1,1200) = 8.21, *p* < 0.01, β = -0.07; see **Table 3**], which persisted when the control variable number of siblings was taken into consideration [Δ*R^2^* = 0.002, Δ*F*(1,1199) = 3.58, *p* < 0.05 one-tailed, β = -0.07]. The results matched the resource dilution model and confirmed that first-born children possess better second language skills than later-born children, regardless of the number of siblings. The competing hypothesis, which assumed that older siblings had a positive effect and ascribed a bridging function to them, was not supported.

**Table 3:** Effect of birth order and older sibling at school age on German language skills.

	German language skills					
Predictors	Model 1^a^			Model 2^b^		
	
	β	*T*	*p*	β	*T*	*p*
Constant		-1.32	0.19		-1.32	0.19
Sex	0.01	0.53	0.60	0.01	0.58	0.57
Age	0.15	6.70	<0.001	0.15	6.57	<0.001
National language	-0.03	-1.04	0.30	-0.02	-0.92	0.36
Number of children’s books	0.36	15.50	<0.001	0.36	15.19	<0.001
Family language	-0.28	-10.82	<0.001	-0.28	-1.89	<0.001
Mothers’ German language skills	0.22	8.29	<0.001	0.22	8.24	<0.001
Fathers’ German language skills	0.07	2.88	<0.01	0.07	2.92	<0.01
Number of siblings				-0.02	-0.53	0.60
Birth order	-0.07	-2.86	<0.01			
Older sibling at school age				-0.05	-1.81	0.07
Δ*R^2^*			0.004			0.002
_c_*_orr_R^2^* (Total)			0.381			0.381

*^a^Step 2 of the hierarchical regression including control variables and Birth order; ^b^Step 2 of the hierarchical regression including control variables and Older sibling at school age; Sex: 1 = male, 2 = female; National language: 1 = countries where German is the national language, 2 = countries where German is not the national language; family language: 1 = exclusively German, 5 = exclusively another language; birth order: 1 = 1 = first-born, 2 = later-born; Older sibling at school age: 1 = no sibling at school-age, 2 = sibling at school age.*

The hypothesis that children with an older sibling at school age had a linguistic advantage over those without an older sibling at school age could not be confirmed. The variable *older sibling at school age* did not significantly explain variance in German skills when the aforementioned control variables and the number of siblings were taken into consideration [Δ*R^2^* = 0.002, Δ*F*(1,1199) = 3.26, *p* = 0.07; β = -0.05; see **Table 3**].

## Discussion

On the basis of a sample of over 1,200 bilingual children, the current study examined whether and how the number of siblings, birth order and presence of an older sibling at school age were associated with second language skills. To do this, two contrasting theoretical approaches, the *resource dilution model* and the *theory of siblings as a facilitator to the local language*, were examined.

As postulated in the first hypothesis, lower second language skills were identified when the number of siblings increased. In line with the resource dilution model, the greatest detriment to second language skills occurred when a first sibling was added. However, the effect size was small. The presence of a second or third sibling did not have statistically significant influence on the second language skills of the target child. Even though this result conforms to the theory and the basic formula 1/*x* (Downey, 1995, 2001), it does to a certain extent contrast with some studies on the effects of siblings among monolingual children. Thus the review of Polit and Falbo (1988) showed that a growing risk of lower linguistic and cognitive performance only exists for families with three or more children, not two. The mixed results regarding the question from which number of children upward detrimental effects exist may be due to families’ financial situation and their position in society. In the current study, immigrant families were examined, most of which have less financial resources at their disposal and a lower status in society than native families (Bundesamt für Statistik [BFS], 2008).

To the authors’ knowledge this is the first study able to examine the assumption that attendance in an early education institution mediates the relationship between the number of siblings and second language skills. According to our study, the resource dilution model can be generalized to families of bilingual children. The inclusion of the mediator *length of attendance in an education institution* reduced the direct effect of the number of siblings on the second language by 84%. This means that with an increasing number of siblings, the hours of attendance in early education institutions decreased. The length of attendance in an early education itself constituted a significant predictor of second language skills explaining 15% in second language skills. Taking the length of attendance in an early education institution into account, the examined variables explained a considerable amount of variance in second language skills (54%).

Although the current study indicates that the resource dilution model can be generalized to the second language acquisition of immigrant children, the significance of different kinds of parental resources may differ for native families and families from immigrant backgrounds. According to Downey (1995), for native families intrapersonal resources such as frequency of conversations within the family and educational aspirations as well as the financial resources invested in educational establishments and learning materials are of central importance. By contrast, for the second language acquisition of immigrant children it appears that financial resources invested in extra-familial learning opportunities are a key component. This may be connected to the lower level of parental skills in the local language, which cannot be passed on directly to the children and instead needs to be bought in the form of education and learning activities (Becker, 2010).

We also examined whether older siblings represented more of an opportunity or a disadvantage for second language acquisition. The current study showed that children *without* older siblings had better second language skills than children with an older sibling, regardless of whether that sibling was already at school or not. As has been ascertained for the first language (Pine, 1995; Berglund et al., 2005; Zambrana et al., 2012), first-born children had a developmental advantage with regard to second language acquisition too. This advantage is small but in a similar range as has been shown in previous studies investigating monolingual children (e.g., Berglund et al., 2005). We can assume that the changes in family constellation following the arrival of a sibling alter the family interaction mechanisms (Strohschein et al., 2008). For example, time for dyadic interaction is reduced (Jones and Adamson, 1987). According to Tomasello and Farrar (1986), it is precisely these dyadic interactions in which the focus of attention does not have to be shared that are particularly valuable for building vocabulary (Mannle et al., 1992). The extent to which this explanation can be applied to multilingual families is unclear. In families where the local language is spoken alongside the native language, mechanisms similar to those in the first language development are conceivable. For families that use only the native language at home, contact with playmates, which is possibly initiated more frequently and consciously by parents for a first-born child than for later-born children, represents a possible explanation.

As the two theories of resource dilution and siblings as a facilitator to the local language refer to different levels (economic versus intrapersonal level), we cannot preclude that both mechanisms occur simultaneously. Even though this study was unable to provide evidence of beneficial effects of older siblings, these may occur nonetheless but may not become apparent due to the existence of negative effects of parental resource dilution.

A further explanation for why no positive effects of older siblings have been found may be the age of the older siblings. Thus it is conceivable that the older siblings of the three-and-a-half-year-old children have only been at school for a short time, and that their own knowledge of the second language is thus too little developed to be of help to their younger siblings (Oller et al., 2011). Possibly, the advantageous effects of older siblings become more evident in older children. Whether older siblings take on a beneficial role for younger children at a later point and then represent a facilitator to the local language and culture is matter that further studies would need to examine.

### Strengths, Limitations, and Future Research

To date, there are only few studies that examine how sibling structure variables are associated with second language acquisition. By researching this connection, the present study provides an important contribution to understanding the development conditions of bilingual children.

One of the study’s main strengths is its sample size, which made it possible to include a great number of control variables, such as sex, age, nationality, family language, and parental German skills. Furthermore, it was possible to test the effect of the number of siblings, something that has frequently posed a problem for previous studies with smaller samples (e.g., Kessler, 1991, cited in Iacovou, 2007).

Despite the strengths, some limitations have to be mentioned. One limitation is the study’s cross-sectional approach, which precludes any statements on effect direction. In their longitudinal study with monolingual families, Rodgers et al. (2000) addressed this question. They showed that it is not the birth order that affects children’s cognitive outcomes, but that parents with lower cognitive skills tend to have more children. In the present study we controlled for parental skills. But despite controlling for these third variables, the question of whether family form really is the reason for lower second language skills remains unanswered and need to be addressed in longitudinal studies.

Another limitation lies in the between-families design. Although sib size is a measure of differences between families, birth order effects are a measure of processes within families (Rodgers et al., 2000). Therefore, the most appropriate way of investigating birth order effects, i.e., comparing elder siblings to their younger siblings, lies in studying sibling pairs in a longitudinal within-families design.

German language skills were measured only by parental report. Even though the parental questionnaire used to assess German skills is highly valid (*r* = 0.84 with a standard German language test), language tests would provide an more precise picture on language skills. Moreover, the studies of Hoff-Ginsberg (1998) and Bornstein et al. (2004) suggest that there may be differences in the effect of sibling order for different measurements of language, e.g., parental questionnaire versus language tests. Thus parental perception of developmental processes and the higher attention paid to developmental changes in first-born children in comparison to later-born children may have been an issue. Thus, it would be interesting to use language test data as dependent variables in future analyses.

Based on the knowledge that siblings take on different roles depending on culture, we assume that the effects and the strength of effects vary across cultures. In cultures that involve older children more strongly in bringing up their younger siblings (Cicirelli, 1994; McGuire and Shanahan, 2010), positive sibling effects on second language acquisition are conceivable. Hence, designing a comparative cultural study would be useful to test this for both first and second language acquisition.

## Conclusion

In sum, the present study was able to show that the resource dilution model can be applied to the second language acquisition of immigrant children. Even though parental resources may not be relevant to first and second language acquisition in the same way, there appears to be an analogy to the first language acquisition in regard to the educational investments made (extra-familial learning opportunities, according to Blake (1981).

Considering that families from immigrant backgrounds have fewer financial resources (Bundesamt für Statistik [BFS], 2008), and that these resources influence the children’s level of development (Mistry et al., 2008), it seems all the more important that immigrant families with many children are financially supported so that their children are offered the best opportunities possible for their academic careers. Given the results of this study, promoting the attendance of early education institutions is an efficient way of achieving this goal.

## Conflict of Interest Statement

The authors declare that the research was conducted in the absence of any commercial or financial relationships that could be construed as a potential conflict of interest.
